# Low apoplastic Na^+^ and intracellular ionic homeostasis confer salinity tolerance upon Ca_2_SiO_4_ chemigation in *Zea mays* L. under salt stress

**DOI:** 10.3389/fpls.2023.1268750

**Published:** 2024-01-03

**Authors:** Moniba Zahid Mahmood, Hamza Ahmad Odeibat, Rafiq Ahmad, Mansour K. Gatasheh, Muhammad Shahzad, Arshad Mehmood Abbasi

**Affiliations:** ^1^ Department of Environmental Sciences, COMSATS University Islamabad, Abbottabad, Pakistan; ^2^ Max Planck Institute for Chemical Ecology, Jena, Germany; ^3^ Department of Biochemistry, College of Science, King Saud University, Riyadh, Saudi Arabia; ^4^ Department of Biotechnology, COMSATS University Islamabad, Abbottabad, Pakistan

**Keywords:** subcellular sodium, calcium silicate, growth, ionic pattern, *Zea mays*, NaCl stress

## Abstract

Salinity is known to have a greater impact on shoot growth than root growth. Na^+^ buildup in plant tissue under salt stress has been proposed as one of the main issues that causes growth inhibition in crops via ionic imbalances, osmotic stress and pH disturbances. However, the evidence for apoplastic Na^+^ buildup and the role of silicon in Na^+^ accumulation at the subcellular level is still enigmatic. The current study focuses on the accumulation of Na^+^ in the apoplast and symplast of younger and older leaves of two maize varieties (Iqbal as salt-tolerant and Jalal as salt-sensitive) using hydroponic culture along with silicon supplementation under short-term salinity stress. Subcellular ion analysis indicated that silicon nutrition decreased Na^+^ concentration in both apoplastic washing fluid and symplastic fluid of maize under salt stress. The addition of silicon under NaCl treatment resulted in considerable improvement in fresh biomass, relative water content, chlorophyll content, and concentration of important subcellular ions (*i.e.*, Ca^2+^, Mg^2+^, and K^+^). Knowledge of subcellular ion analysis is essential for solving the mechanisms underlying vital cellular functions e.g. in the current study, the soluble Na^+^ concentration in the apoplast of older leaves was found to be significantly greater (36.1 mM) in the salt-sensitive variety under NaCl treatment, which was 42.4% higher when compared to the Na^+^ concentration in the salt-tolerant variety under the same treatment which can influence permeability of cell membrane, signal transduction pathways and provides insights into how ion compartmentalization can contributes to salt tolerance. Calcium silicate enrichment can contribute to increased growth and improved ionic homeostasis by minimizing leaf electrolyte leakage, improving mechanical functions of cell wall and reducing water loss, and improved photosynthetic function. In current investigation, increased water content and intracellular ionic homeostasis along with reduced concentration of Na^+^ in the maize leaf apoplast suggest that calcium silicate can be used to ameliorate the adverse effects of salt stress and obtain yield using marginal saline lands.

## 1 Introduction

Increased land degradation and environmental changes pose significant challenges due to their far-reaching impacts on ecosystems services, biodiversity, food security, and human well-being. Land degradation via salinity, harm plant growth, productivity, and ultimately yield, causing significant economic losses and a food crisis (Gupta, 2019; Derakhshani et al., 2020). Almost all crops grow poorly under saline environments, but their salt tolerance and rates of growth reduction vary widely among species of plants (Jesmin et al., 2023; Munir et al., 2022). Understanding and utilizing this variability is essential not only for selecting and breeding salt-tolerant crop varieties but also for the sustainable use of land resources. Salinity is a significant issue, particularly in arid and semiarid areas, due to inadequate rain, high temperatures, heavy evapotranspiration, and inappropriate soil management practices (Hassani et al., 2021). Salinity already affects more than 20% of agricultural land worldwide, and its extent is expanding, which is a threat to global agriculture considering the increasing population (Hossain, 2019). The expansion of saline-affected lands hinders the ability to produce enough food to meet the needs of an expanding population (Lam et al., 2022). As the world’s population continues to grow, addressing the issue of salinity becomes essential to achieving global food security, alleviating hunger, and ensuring that nutritious and diverse food is available to all. With an addition of 10% of land area becoming salinized annually, it is predicted that salinity will affect 50% of arable land by 2050 (Kumar and Sharma, 2020; Muthuraja and Muthukumar, 2022). Considering projected growth in both population and food security, it is imperative to manage and find solutions to increase agricultural productivity and economic yields using saline lands (Javed et al., 2022).

The agriculture sector in Pakistan is the backbone of the nation’s economy, contributing in gross domestic product (GDP) by up to 22.7% and meeting people’s livelihood employment needs (GoP, 2023). Therefore, the issue of salinity is particularly significant to Pakistan where agriculture plays a vital role in the economy and provides employment to a large portion of the population (Qureshi and Perry, 2021). After rice and wheat, maize is regarded as the 3^rd^ most significant cereal crop and a staple food for a large proportion of the world’s population (Ali et al., 2018). In Pakistan, maize is the 3^rd^ important cereal crop and it contributes 0.7 percent to GDP. Maize was cultivated on 1.65 million hectares of land in 2021–2022, and overall yield was 10.6 million tons (GoP, 2023). Salinity disrupts maize cultivation by delaying seed germination, root growth, nutrient uptake, and overall plant development. Elevated salt levels in the soil reduced water absorption by plant roots, leading to water stress and reduced nutrient availability (Mumtaz et al., 2021). These factors collectively diminish maize yields, impacting both food security and economic prosperity. According to Rasool et al. (2013), maize production is limited in Pakistan as a result of salinity, shortage of high-quality water, and high soil pH. However, soil salinity in Pakistan affects nearly 6 million hectares of agricultural land. In many areas, maize yields are lower than their potential due to saline soil conditions (Syed et al., 2021). This not only reduces the quantity of maize produced but also affects its quality, as salinity can lead to nutrient imbalances and poor grain formation (Javed et al., 2022). Therefore, the challenges posed by salinity on the agriculture in Pakistan, particularly on maize cultivation, is a significant concern with far-reaching consequences which include food security, economic stability, and rural livelihoods. As Pakistan strives to feed its growing population and strengthen its agricultural sector, addressing soil salinity through innovative solutions and sustainable practices becomes imperative to ensure the resilience of maize cultivation and overall food production.

In general maize is classified as a salt-sensitive crop. Salinity stress has negative impacts on morphological attributes, physiological mechanisms, and biochemical changes, that lead to reduction in germination of seeds, fresh and dry biomass, photosynthesis, and the accumulation of mineral nutrients (Naveed et al., 2020). At the first stage of salt stress in plants, a rapid decline in shoot growth occurs as a result of osmotic stress (Munns and Tester, 2008), resulting in a decline in the amount of water available and photosynthesis (Soni et al., 2021), and at the later stage by ionic stress, leading to nutritional mineral deficiencies and ion-specific toxicity (Basu et al., 2021).

High salinity decreases the external osmotic pressure and is thought to increase the amount of Na^+^ in shoots, which eventually impacts plant growth by adversely affecting nutrient transport, metabolic activities, stomatal conductance, photosynthetic apparatus, carbon fixation, enzyme activities etc. (Balasubramaniam et al., 2023). It has been suggested that shoots are more prone to sodium ions than roots (Tester and Davenport, 2003). Under highly saline soil, crops can face extensive osmotic and metabolic stress in the shoot, which can affect their normal functions (*i.e.*, stomatal closure, lessens photosynthesis, and prevents the expansion of roots and developing leaves) through the increased concentration of Na^+^ (Van Zelm et al., 2020). The intricate interactions of poor water relations, nutritional imbalance, plant hormones, and the specific ion effect on plant growth in salt-affected environments ultimately result in decreased plant growth and yield reduction. Earlier study (Apse et al., 1999) suggested that ion toxicity effects can be alleviated by the active exclusion of cytosolic Na^+^ to the apoplast and vacuole. However, Na^+^ accumulation above a certain threshold can increase osmotic pressure and damage cellular organelles (Sathee et al., 2022).

Silicon (Si) is the 2^nd^ most common element in the crust of the earth, and plants often contain Si levels that are comparable to those of macronutrients (Kang et al., 2015). The growth of plants during abiotic stress has been shown to benefit from exogenous Si treatment (Balakhnina et al., 2015; Waheed et al., 2021). Si application under NaCl stress resulted in reduced uptake of sodium ions in total shoots and more biomass production in contrast with the control in crops, e.g., barley, *Phaseolus vulgaris* L., and *maize* (Liang et al., 2005; Zuccarini, 2008; Sattar et al., 2016). Si ability to mitigate abiotic stress is attributed to several mechanisms affecting plant responses to stress such as salinity. One prominent mechanism is the reinforcement of cell walls through the deposition of silica which forms a mechanical barrier, stimulating cell walls and interpreting them less permeable to ions, thereby reducing sodium influx in saline conditions and preventing cell dehydration during salt stress (Vandegeer et al., 2021). Under stress Si aids in the accumulation of osmolytes, such as proline and soluble sugars, which sustain cell turgor and restrict water loss. This osmotic regulation enhances the plant’s ability to withstand water deficits and osmotic stress which is generally induced by increased salt concentrations (Ashfaq et al., 2022). However, studies dealing with Na^+^ accumulation at the subcellular level and its contribution to growth along with Si treatment under salt stress to understand the mechanism behind stress alleviation are limited. Therefore, the current investigation hypothesize the comparative accumulation of Na^+^ in the subcellular leaf parts of two maize varieties different in salt tolerance, its impacts on growth reduction, and the application of calcium silicate to investigate its ameliorative role on the possible negative consequences of leaf apoplastic Na^+^ under salt stress.

## 2 Materials and methods

### 2.1 Cultivation of plants

Seeds of maize (*Zea mays* L.) were obtained from Cereal Crops Research Institute (CCRI), Pirsabak, Nowshera, Pakistan. Salt-tolerant (*i.e.*, Iqbal) and salt-sensitive (*i.e.*, Jalal) maize varieties were used for experimentation. Initially, maize seeds were soaked in 0.5 mM calcium sulfate (CaSO_4_) for 12 h with continuous aeration. The seeds were germinated by using the sandwich method at 28°C in the dark. Seedlings were placed in the light after 4 days. On the 5^th^ day, seedlings were transferred to hydroponics (*i.e.*, plastic pots with 20 L capacity and with continuous aeration) with nutrient solution at 1/4^th^ concentration. After the 2^nd^ and 4^th^ days of transfer to hydroponics, the nutrient solution concentration was raised to half‐ and full‐strength, respectively. The full concentration of nutrient solution was 0.2 mM KH_2_PO_4_, 5 µM H_3_BO_3_, K_2_SO_4_, 2 µM MnSO_4_, 2 mM Ca (NO_3_)_2_, 0.5 µM ZnSO_4_, 0.5 mM MgSO_4_, 0.3 µM CuSO_4_, 0.2 mM Fe-EDTA, and 0.01 µM (NH_4_)Mo_7_O_24_, as mentioned in Witzel et al. (2011). To prevent nutrient deficits, the nutrition solution was replaced twice weekly. The experiment was conducted under controlled conditions with a photoperiod of 14 h, an average day/night temperature of 28/18°C and approximately 60 ± 5% relative humidity.

### 2.2 NaCl and Ca_2_SiO_4_ treatments

NaCl treatments were initiated after attaining full strength of the nutrient solution, and the observed levels of NaCl doses were 100 mM NaCl for saline treatments and 1 mM NaCl for the control. Plants were gradually acclimatized through daily increments of 25 mM NaCl until 100 mM NaCl was achieved. With the addition of NaCl, calcium silicate was also added with different treatments in the nutrient solution. Overall, four treatments were used, NaCl (1mM) - Control; NaCl (100 mM) - NaCl; Ca_2_SiO_4_ (1 mM) - Ca_2_SiO_4_; NaCl (100 mM) + Ca_2_SiO_4_ (1 mM) – NaCl + Ca_2_SiO_4_ in the pot. A complete randomized block design was used to perform the experiment, where each treatment was run with four technical replicates and with at least fifteen biological replicates. Harvesting of maize plants was started after 21 days for further analysis.

### 2.3 Growth parameters, chlorophyll content and relative water content

After 21 days, five biological replicates of each treatment were harvested, and the following morphological parameters were measured. Plants were harvested and divided into older and younger parts. The fresh weight (FW) of each plant fraction was separately weighed; however, for the dry weight (DW) samples were first oven-dried at 60°C for 72 hours until a constant weight was achieved by using an analytical weigh balance (Model no. JJ224BC). The chlorophyll content of older and younger leaves of maize plants was determined by using a CCM 200+ meter (Shah et al., 2017). To analyze the RWC [%], the turgid weight (TW) was obtained, and later, the following equation was used for calculations.


$$\text{RWC}[\%]=\frac{\text{FW}-\text{DW}}{\text{TW}-\text{DW}}\times 100$$

### 2.4 Ionic analysis in plant samples

To determine the total ion concentration in the leaves and roots of maize, dried samples were powdered manually by using a ceramic mortar and pestle. The 100 mg weighed samples were then placed in ceramic pots and ashes in a furnace at 520°C for 5 hours. After that, 2 ml of 4 M HNO_3_ was added to each sample and gently mixed every half hour for a total of three hours to create a suspension. Later, 8 ml of distilled water was added to a final volume of 10 ml. The suspension was then filtered through Whatman filter paper No. 21 into vials. Thereafter, Na^+^, K^+^, Ca^2+^, and Mg^2+^ concentrations were examined in the filtrate by using an atomic absorption spectrophotometer (AAS) (AAnalyst 700, Perkin Elmer, USA).

### 2.5 Extraction of apoplastic washing fluid and symplastic fluid from maize leaves

AWF was obtained by using an infiltration-centrifugation method with minor modifications (Lohaus et al., 2001). For infiltration, leaves that had no visible salt stress injury symptoms were excised after 16 days of reaching the maximum salinity level, *i.e.*, 100 mM NaCl. The leaves were cut into 5.5 cm long small segments, weighed, and later carefully washed using deionized water. For the infiltration process, leaf segments were placed in plastic syringes (60 ml) and filled with 50 ml of deionized water. Later, air was removed, and by pulling the plunger and creating an approximate reduced pressure of approximately 20 kPa, leaves were infiltrated. Intact segments of the leaves were blotted dry, weighed, and later gently placed in a 10-ml plastic vessel, and this whole was further fitted in 50 ml Falcon tubes and immediately centrifuged at 5°C at 400 × g for 5 min. The collected solution was weighed and designated AWF. The remaining leaf tissue after the extraction of AWF was frozen in liquid nitrogen and later thawed and centrifuged at 715 g for 5 min for the extraction of the cell sap, and the collected fluid was referred to as the SF.

### 2.6 Ionic analysis in the AWF and SF of maize leaves

The AWF and SF of each sample were oven-dried at 80°C for 4 hours. One milliliter of 4 M HNO_3_ was added and gently mixed every half hour for a total of three hours to create a suspension. Later, 2 ml of deionized water was added, and a final volume of 3 ml was achieved. To analyze Na^+^, K^+^, Ca^2+^, and Mg^2+^, an atomic absorption spectrophotometer (Analyst 700, Perkin Elmer, USA) was used, as suggested by A.O.A.C (association of official analytical chemist) protocol (Helrich, 1990). The unit ‘mM’ was used to present the concentration of cations in the leaf apoplast of maize plants.

### 2.7 Statistical analysis

The data were analyzed using two-way analysis of variance (ANOVA) and Tukey’s test, which were performed using the univariate general linear model, which yielded significant differences between means that were distributed normally and compared at P ≤ 0.05. These differences were labeled using small letters that were positioned at the top of each bar. The data are presented as the means ± standard errors (SE). SPSS, the Statistical Package for the Social Sciences, version 23.0, was used to analyze the data.

## 3 Results

### 3.1 Fresh biomass production in maize under salt stress

**Figure 1:**
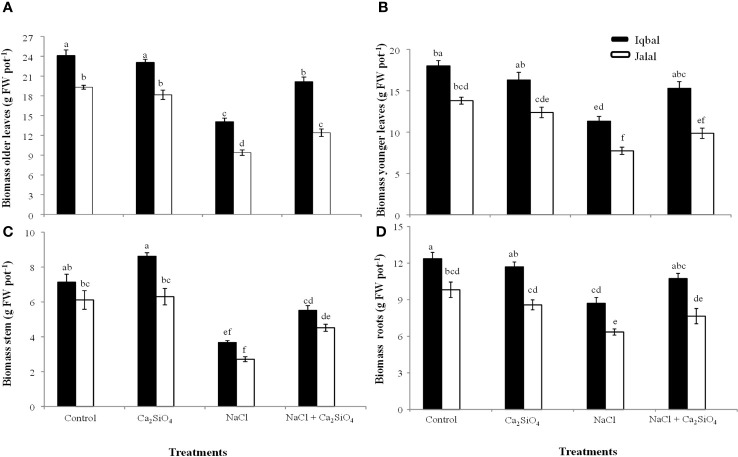
Influence of Control, Ca_2_SiO_4_, NaCl, and NaCl + Ca_2_SiO_4_ on the fresh biomass (g pot^-1^) of older leaves **(A)**, younger leaves **(B)**, stem **(C)**, and roots **(D)** of maize varieties (*i.e.*, black bars represent Iqbal while white bars represent Jalal). The result presented are means and standard error (±), however, differences between the treatments at P ≤ 0.05, and n≥5 are indicated by different letters.

The greater fresh biomass in older leaves was observed in the control in the Iqbal variety (24.1 g pot^-1^), while the lower was noticed in the NaCl treatment in the Jalal variety (9.4 g pot^-1^). NaCl treatment significantly reduced the fresh biomass of older leaves in both varieties; however, when compared to the NaCl treatment, the addition of Ca_2_SiO_4_ with NaCl resulted in 30% and 24% increases in Iqbal (20.1 g pot^-1^) and Jalal (12.4 g pot^-1^), respectively (**Figure 1A**). Our findings related to fresh biomass in younger leaves showed that the addition of Ca_2_SiO_4_ with NaCl treatment resulted in 26% and 22% increases in the Iqbal variety (15.3 g pot^-1^) and Jalal variety (9.9 g pot^-1^), respectively, compared to NaCl alone (**Figure 1B**).

The greater fresh biomass in the stem was observed under Ca_2_SiO_4_ (8.6 g pot^-1^) followed by the control (7.1 g pot^-1^) in the Iqbal variety, while the lowest fresh biomass of the stem was observed in Jalal (2.7 g pot^-1^) in NaCl. However, when compared to the NaCl treatment alone, the fresh biomass production of stems was increased with the introduction of Ca_2_SiO_4_ along with NaCl treatment, which was 33% in the Iqbal variety (5.5 g pot^-1^) and 40% in the Jalal variety (4.5 g pot^-1^) (**Figure 1C**). In the roots of maize plants, the greater fresh biomass was found in the Iqbal variety (12.4 g pot^-1^) in the control, while the lower fresh biomass was recorded in the Jalal variety (6.4 g pot^-1^) under saline condition. The addition of Ca_2_SiO_4_ with NaCl treatment resulted in increased fresh biomass production of roots, which was 19% in Iqbal (10.7 g pot^-1^) and 17% in the Jalal variety (7.7 g pot^-1^) (**Figure 1D**).

### 3.2 RWC in maize leaves

In older leaves of maize plants, the lowest RWC was measured in the Jalal variety (48.3%) under NaCl treatment, while the highest was recorded in Iqbal, *i.e.*, 84.6% in Ca_2_SiO_4_ (**Table 1**). When compared to the RWC in older leaves in the NaCl treatment, maize varieties showed increased RWC with a combination of Ca_2_SiO_4_ and NaCl, which were 14.5% in the Iqbal variety and 11.8% in the Jalal variety. In younger maize leaves, the lowest RWC of 40.3% was observed in the Jalal variety under NaCl treatment, while the highest was found in the Iqbal variety (78.2%) under Ca_2_SiO_4_ (**Table 1**). Moreover, compared to salt stress alone, when Ca_2_SiO_4_ was added along with NaCl, the RWC in younger leaves increased by approximately 22% and 19% in the Iqbal and Jalal varieties, respectively.

**Table 1:** Estimated chlorophyll content (SPAD value) and relative water content (%) in older and younger leaves of maize varieties.

Treatments	Estimated chlorophyll content (SPAD Value)				Relative water content (RWC) %			
	Older leaves		Younger leaves		Older leaves		Younger leaves	
	Iqbal	Jalal	Iqbal	Jalal	Iqbal	Jalal	Iqbal	Jalal
**Control**	51.9 ± 1.14^bc^	49.1 ± 0.84^c^	34.9 ± 1.47^b^	31.8 ± 0.98^b^	80.5 ± 1.81^a^	77.6 ± 0.73^ab^	76.2 ± 1.71^a^	70.4 ± 1.93^ab^
**Ca_2_SiO_4_**	58.8 ± 0.78^a^	55.2 ± 0.77^ab^	42.6 ± 1.45^a^	41.6 ± 1.02^a^	84.6 ± 1.07^a^	79.8 ± 1.20^ab^	78.2 ± 0.76^a^	72.1 ± 1.45^ab^
**NaCl**	28.9 ± 1.17^e^	20.4 ± 0.35^f^	20.5 ± 1.77^c^	12.8 ± 1.06^d^	62.2 ± 0.88^c^	48.4 ± 2.43^d^	50.9 ± 1.14^c^	40.3 ± 2.87^d^
**NaCl+Ca_2_SiO_4_**	41.7 ± 0.85^d^	26.2 ± 1.81^e^	30.5 ± 0.92^b^	16.5 ± 1.00^cd^	72.8 ± 1.54^b^	54.8 ± 2.18^d^	64.5 ± 2.36^b^	49.7 ± 3.58^cd^

Different letter (a-d) indicate significant variations at p ≤ 0.05.

### 3.3 Estimated chlorophyll contents in maize leaves

Chlorophyll contents were higher in both younger and older leaves in the Iqbal variety than in the Jalal variety in all the treatments (**Table 1**). Moreover, in comparison to the control, NaCl significantly reduced the chlorophyll contents in both Iqbal and Jalal varieties older and younger leaves. However, in comparison to the NaCl treatments in older leaves, the chlorophyll contents were significantly higher (≥1.3-fold) in both the Iqbal and Jalal varieties (**Table 1**). In younger leaves, the addition of Ca_2_SiO_4_ with NaCl treatment resulted in significantly higher chlorophyll contents in the Iqbal variety (1.4-fold) when compared to NaCl treatment alone.

### 3.4 The concentration of ions in *Zea mays* L. leaves

**Figure 2:**
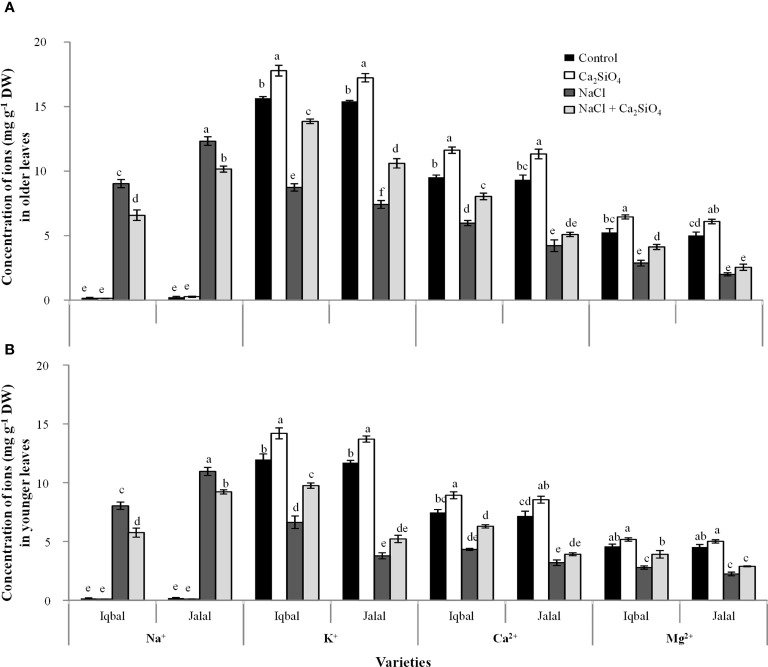
Concentration (mg g^-1^ DW) of ions (*i.e.*, Na^+^, K^+^, Ca^2+^, and Mg^2+^) in older leaves **(A)**, and younger leaves **(B)** of maize varieties (*i.e.*, Iqbal and Jalal) as influenced by Control, Ca_2_SiO_4_, NaCl, and NaCl + Ca_2_SiO_4_. The results displayed are means and standard error (±), however, differences between the treatments at P ≤ 0.05, and n≥5 are indicated by different letters.

In older leaves, when a comparison was made among treatments, the Na^+^ concentration was noticed greater under NaCl in both Iqbal (9.1 mg g^-1^ DW) and Jalal (12.3 mg g^-1^ DW) varieties (**Figure 2A**). However, a decrease in Na^+^ concentration with the introduction of Ca_2_SiO_4_ with NaCl treatment was more pronounced in the Iqbal variety (27%) than in the Jalal variety (17%) compared to NaCl alone (*i.e.*, 100 mM). In older leaves, when a comparison was made among treatments, the greater K^+^ concentration was found in the Ca_2_SiO_4_ treatment alone (17.7 mg g^-1^ DW). When we compared the increase in K^+^ concentration (*i.e.*, with respect to NaCl treatment alone) with the addition of Ca_2_SiO_4_ with NaCl treatment (100 mM), the Iqbal variety resulted in a 37% increase compared to the Jalal variety (30%). A significant increase (25.1%) in Ca^2+^ concentration with the addition of Ca_2_SiO_4_ with NaCl treatment was noticed in the Iqbal variety when compared to NaCl treatment alone. In comparison to the control, the Mg^2+^ concentration was significantly reduced with NaCl treatment in both the Iqbal and Jalal varieties; however, in comparison to NaCl treatment alone, the supplementation of Ca_2_SiO_4_ with NaCl treatment resulted in a 30.4% increase in the Iqbal variety and a 21.3% increase in the Jalal variety (**Figure 2A**).

In younger leaves of maize plants, the Na^+^ concentration was greater in the NaCl treatment in the Jalal variety (10.9 mg g^-1^ DW) (**Figure 2B**). In comparison to the Na^+^ concentration in the NaCl treatments, the addition of Ca_2_SiO_4_ to the NaCl treatment led to a significantly reduced Na^+^ concentration in younger leaves of the Iqbal variety (28%). In comparison to the control, in younger leaves of the Iqbal variety, NaCl resulted in reduced K^+^, Ca^2+^, and Mg^2+^ concentrations by 45%, 42%, and 39%, respectively. The addition of Ca_2_SiO_4_ with NaCl treatment improved K^+^, Ca^2+^, and Mg^2+^ concentrations in the Iqbal variety by 32%, 31 and 29%, respectively, compared to NaCl treatments alone. In comparison to the control, in younger leaves of the Jalal variety, NaCl resulted in reduced K^+^, Ca^2+^, and Mg^2+^ concentrations by 68%, 55%, and 51%, respectively. However, the addition of Ca_2_SiO_4_ with NaCl treatment improved K, Ca, and Mg concentrations in the Jalal variety by 28%, 18%, and 23%, respectively, compared to NaCl treatments alone.

### 3.5 Ionic concentration in the extracted AWF of maize leaves

**Figure 3:**
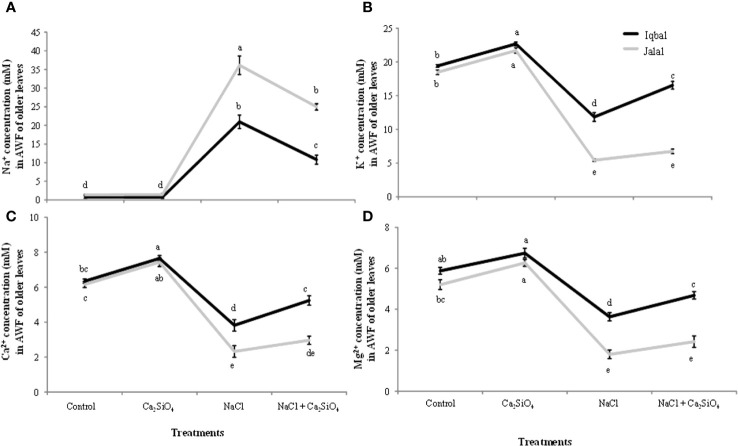
Influence of Control, Ca_2_SiO_4_, NaCl, and NaCl + Ca_2_SiO_4_ on the Na^+^ concentration (mM) **(A)** K^+^ concentration (mM) **(B)** Ca^2+^ concentration (mM) **(C)** and Mg^2+^ concentration (mM) **(D)** in the apoplastic washing fluid (AWF) of older leaves of maize varieties (*i.e.*, Iqbal and Jalal). The results displayed are means and standard error (±), however, differences between the treatments at P ≤ 0.05, and n≥5 are indicated by different letters.

The soluble Na^+^ concentration in the extracted AWF in older leaves of maize plants was found to be significantly greater in the Jalal variety at 36.1 mM in the NaCl treatment, which was 42.4% higher when compared to the Na^+^ concentration in the Iqbal variety under the same treatment (**Figure 3A**). Our findings with the addition of Ca_2_SiO_4_ with NaCl treatment showed that the greater soluble Na^+^ concentration in the extracted AWF in older leaves of maize plants was 24.9 mM in the Jalal variety, which was 56.6% higher than the Na^+^ concentration in the Iqbal variety under the same treatment (**Figure 3A**). In older leaves, when comparison was made among varieties, the Iqbal variety showed higher K^+^, Ca^2+^ and Mg^2+^ concentrations in the AWF compared to the Jalal variety in all the treatments. In comparison to the control, in older leaves of the Jalal variety, NaCl resulted in reduced K^+^, Ca^2+^, and Mg^2+^ concentrations by 71%, 62%, and 65%, respectively. However, the addition of Ca_2_SiO_4_ with NaCl treatment resulted in improved K^+^, Ca^2+^, and Mg^2+^ concentrations in the Jalal variety by 19%, 22%, and 26%, respectively, compared to NaCl treatments alone. In older leaves of the Iqbal variety, in comparison to the control, NaCl resulted in reduced K^+^, Ca^2+^, and Mg^2+^ concentrations by 39%, 40%, and 38%, respectively. The addition of Ca_2_SiO_4_ with NaCl treatment improved K^+^, Ca^2+^, and Mg^2+^ concentrations in the Iqbal variety by 28%, 27% and 22%, respectively, compared to NaCl treatments alone.

**Figure 4:**
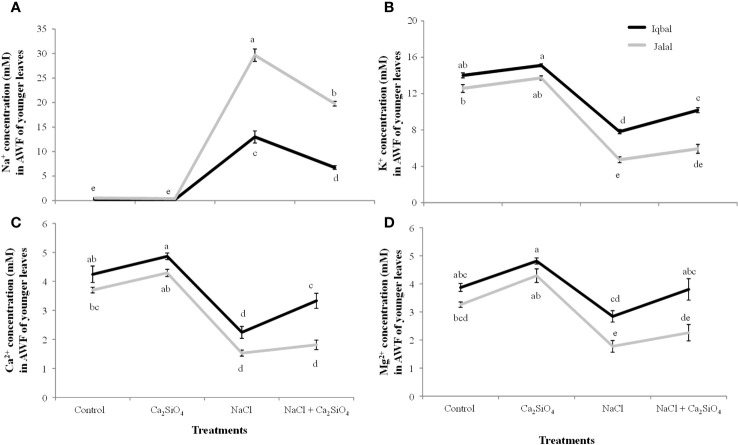
Na^+^ concentration (mM) **(A)** K^+^ concentration (mM) **(B)** Ca^2+^ concentration (mM) **(C)** and Mg^2+^ concentration (mM) **(D)** in the apoplastic washing fluid (AWF) of younger leaves of maize varieties (*i.e.*, Iqbal and Jalal) as influenced by Control, Ca_2_SiO_4_, NaCl, and NaCl + Ca_2_SiO_4_. The results displayed are means and standard error (±), however, differences between the treatments at P ≤ 0.05, and n≥5 are indicated by different letters.

In comparison to the ionic (Na^+^, K^+^, Ca^2+^, and Mg^2+^) concentrations in the AWF of older leaves, all the ions showed reduced cAWF of younger leaves in both Iqbal and Jalal maize varieties (**Figures 3**, **4**). Significantly higher soluble Na^+^ concentration (29.6 mM) was found in the AWF of younger leaves of the Jalal variety under NaCl stress, which was 73% higher when compared to the Iqbal variety under the same treatment (**Figure 4A**). The influence of Ca_2_SiO_4_ with NaCl treatment resulted in reduced soluble Na^+^ concentrations in both the Iqbal (48%) and Jalal (33%) varieties. All the other ions (K^+^, Ca^2+^, and Mg^2+^) concentrations were significantly reduced by 43%, 47%, and 27% in Iqbal and 63%, 59%, and 46% percent in the Jalal variety under NaCl treatment alone when compared to their respective controls in the AWF of younger leaves (**Figures 4B–D**). Moreover, with Ca_2_SiO_4_ addition along with NaCl treatment, ionic concentrations (K^+^, Ca^2+^, and Mg^2+^) were improved by 23%, 33%, and 25% in the AWF of younger leaves of the Iqbal variety. However, in the Jalal variety, improvement in the ionic concentration (K^+^, Ca^2+^, and Mg^2+^) was 20%, 16%, and 22% percent in the AWF of younger leaves with a supplement of Ca_2_SiO_4_ with NaCl.

### 3.6 Ionic concentration in the extracted SF of maize leaves

**Figure 5:**
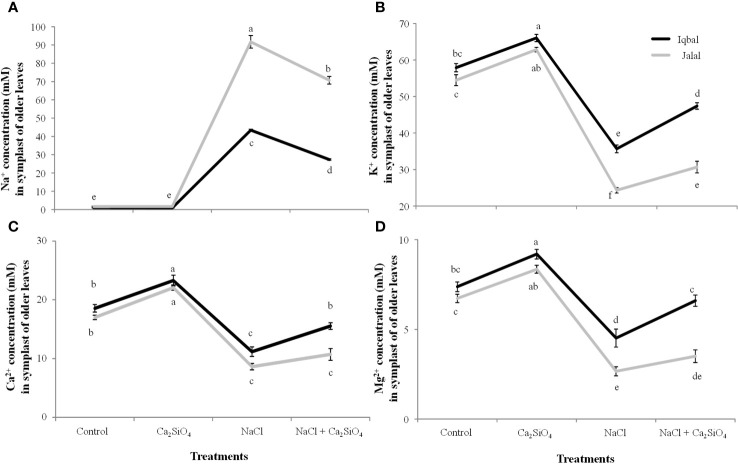
Influence of Control, Ca_2_SiO_4_, NaCl, and NaCl + Ca_2_SiO_4_ on the Na^+^ concentration (mM) **(A)** K^+^ concentration (mM) **(B)** Ca^2+^ concentration (mM) **(C)** and Mg^2+^ concentration (mM) **(D)** in the symplast of older leaves of maize varieties (*i.e.*, Iqbal and Jalal). The results displayed are means and standard error (±), however, differences between the treatments at P ≤ 0.05, and n≥5 are indicated by different letters.

In the extracted SF of older leaves of maize, a significantly greater Na^+^ concentration was found in the Jalal variety (91.7 mM) in the NaCl treatment, which was 53% higher in contrast to the Na^+^ concentration in the Iqbal variety (**Figure 5A**). In comparison to NaCl alone, a combination of Ca_2_SiO_4_ and NaCl resulted in significantly reduced Na^+^ concentrations in the extracted SF of older leaves of the Iqbal (37%) and Jalal (23%) varieties (**Figure 5A**). K^+^, Ca^2+^ and Mg^2+^ concentrations were 32%, 28%, and 42% higher in the extracted SF of older leaves of Iqbal varieties when compared to Jalal varieties under NaCl treatment (**Figures 5B–D**). Improvements in the ionic concentrations (K^+^, Ca^2+^, and Mg^2+^) in the extracted SF of older leaves were 25%, 28%, and 32% in the Iqbal variety and 21%, 20%, and 24% in the Jalal variety when compared to their respective NaCl treatments alone (**Figures 5B–D**).

**Figure 6:**
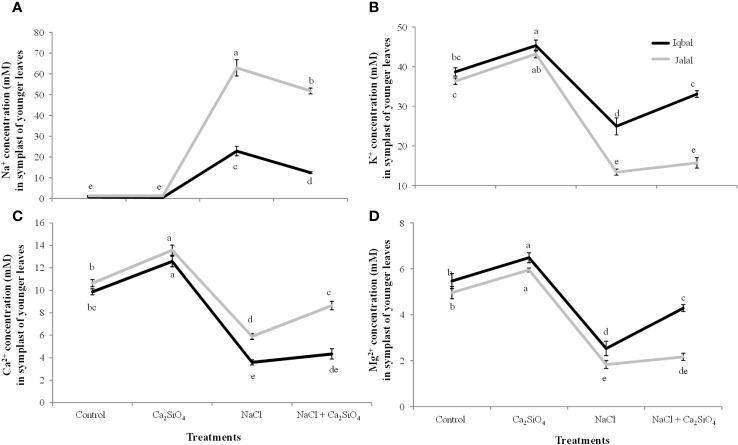
Na^+^ concentration (mM) **(A)** K^+^ concentration (mM) **(B)** Ca^2+^ concentration (mM) **(C)** and Mg^2+^ concentration (mM) **(D)** in the symplast of younger leaves of maize varieties (*i.e.*, Iqbal and Jalal) as influenced by Control, Ca_2_SiO_4_, NaCl, and NaCl + Ca_2_SiO_4_. The results displayed are means and standard error (±), however, differences between the treatments at P ≤ 0.05, and n≥5 are indicated by different letters.

In the extracted SF of younger maize leaves, lower ionic concentrations (Na^+^, K^+^, Ca^2+^, and Mg^2+^) were found in both Iqbal and Jalal varieties compared to the older leaves (**Figures 5**, **6**). Under NaCl treatment, a significantly higher Na^+^ concentration (62.9 mM) was observed in the extracted SF of younger maize leaves in the Jalal variety, which was 63.7% higher than that in the Iqbal variety under the same treatment (**Figure 6A**). In comparison to NaCl treatment alone, a significant reduction was observed in the Na^+^ concentration in the extracted SF of younger maize leaves of both Iqbal 46% and Jalal 18% when Ca_2_SiO_4_ was supplemented with NaCl. In the extracted SF of younger leaves, K^+^, Ca^2+^, and Mg^2+^ concentrations were reduced by 36%, 45%, and 54% in the Iqbal variety and 63%, 64%, and 63% in the Jalal variety under NaCl treatment alone compared to their respective controls (**Figures 5B–D**). Improvements in the ionic concentrations (K^+^, Ca^2+^, and Mg^2+^) in the extracted SF of younger leaves were 25%, 32%, and 41% in the Iqbal variety and 15%, 17%, and 16% in the Jalal variety when compared to their respective NaCl treatments alone (**Figures 6B–D**).

**Figure 7:**
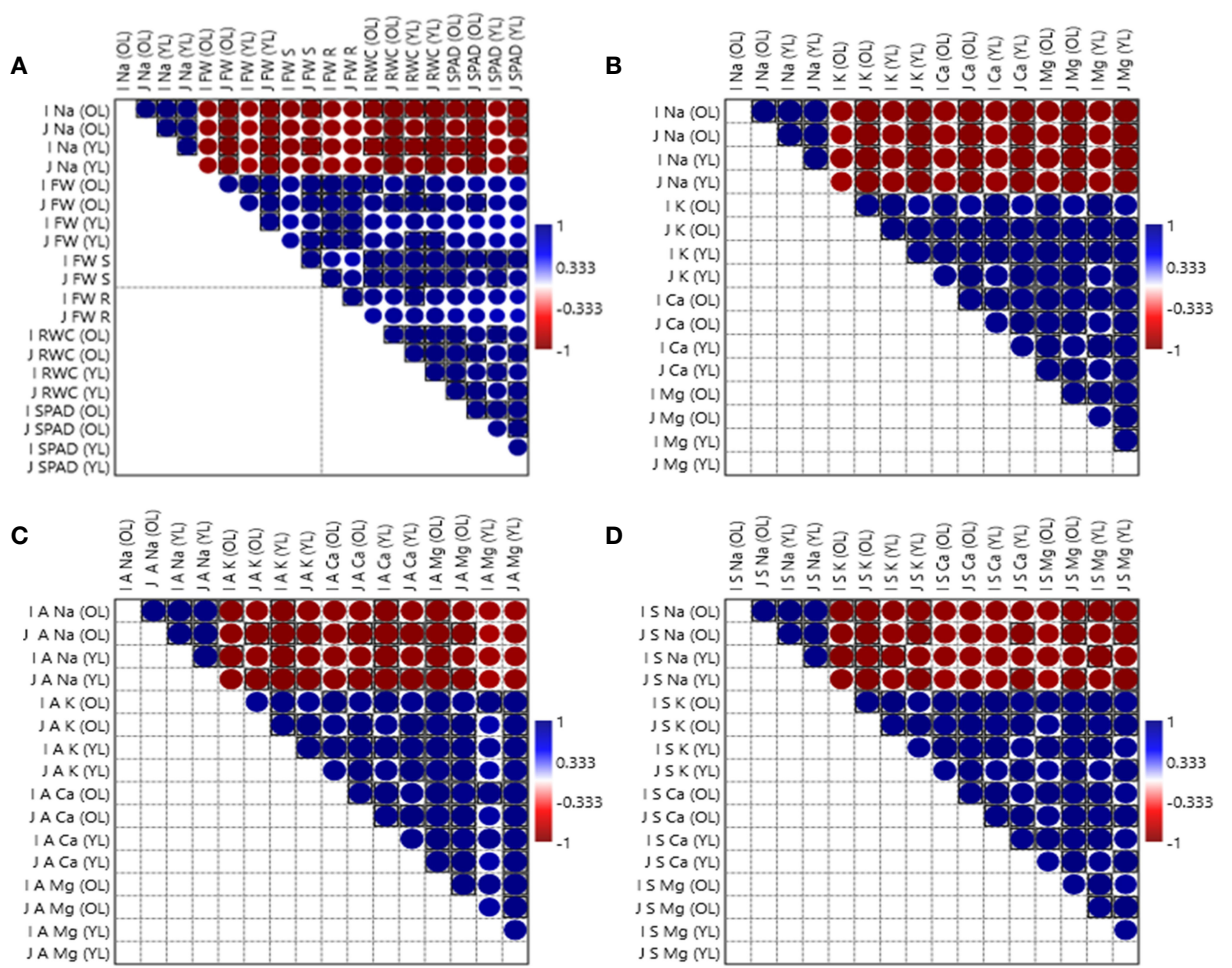
Correlation between Na^+^ concentration in each fraction of *Zea mays* L. *i.e.*, ‘OL’ for older leaves ‘YL’ for younger leaves ‘A’ for apoplast and ‘S’ for symplast in Iqbal as salt-tolerant variety denoted with ‘I’ and for Jalal as salt-sensitive variety denoted with ‘J’ with all other studied parameters *i.e.*, ‘FW’ fresh weight, ‘RWC’ relative water content, ‘SPAD’ chlorophyll content **(A)**, ‘K’ K^+^ concentration, ‘Ca’ Ca^2+^ concentration, ‘Mg’ Mg^2+^ concentration in total shoot **(B)**, in apoplast **(C)** and in symplast **(D)** of *Zea mays* L.

Moreover, correlation coefficient matrixes as illustrated in **Figure 7**, indicate that Na^+^ concentration in each fraction of *Zea mays* L. *i.e.*, older leaves, younger leaves, apoplast and symplast in salt-tolerant and salt-sensitive varieties had strong negative associations with all other studied parameters *i.e.*, fresh weight, relative water content, chlorophyll content (**Figure 7A**), K^+^ concentration, Ca^2+^ concentration, Mg^2+^ concentration in total shoot (**Figure 7B**), in apoplast (**Figure 7C**) and in symplast (**Figure 7D**) of *Zea mays* L.

## 4 Discussion

### 4.1 Influence of salinity on maize biomass production

The osmotic phase of salt stress causes a rapid reduction in plant growth. Elevated salt levels create an osmotic imbalance, reducing the availability of water to plant roots which leads to water stress and decreased water uptake and overall plant reduced turgor pressure needed for cell expansion and growth. Moreover, excessive Na^+^ accumulate in plant tissues under salinity stress and interferes with the uptake of many cations resulting in nutrient imbalance which can disturb the regulation of cell homeostasis. Therefore, excessive amounts of salts in the growth medium lead to disturbances in plant physiological functions, including biochemical and gas exchange properties and ionic concentrations, which directly affect plant growth (Kamran et al., 2019; Mann et al., 2020). Earlier studies, (Munns, 1993; Fortmeier and Schubert, 1995) proposed that older leaves die first as a result of the fast build-up of high Na^+^ when vacuoles are unable to sequester incoming salts, especially in crop plants. The vacuole sequestration of Na^+^ is a critical adaptive strategy for plants under salt stress as it helps maintain cellular ion homeostasis, osmotic balance, and overall plant health. By prioritizing the protection of younger tissues and preserving vital cellular processes like photosynthesis, plants can continue to grow and thrive in saline environments. This process is a key factor in the salt tolerance of many plant species. In the current study, compared to the control, fresh biomass of the salt-sensitive variety resulted in a 51% reduction in older leaves and a 43% reduction in younger leaves under NaCl, while the salt-tolerant variety presented a 41.6% reduction in older leaves and a 37.1% reduction in younger leaves (**Figures 1A, B**). The percentage reduction in biomass serves as an indicator of a plant’s salt tolerance. A higher reduction in biomass suggests that the plant is more vulnerable to salt stress, while a lower reduction indicates a higher level of salt tolerance. This information is necessary for evaluating the suitability of plant varieties for cultivation in saline environments. Salinity has a strong influence on growth processes, and biomass yield and growth rate are considered reliable indicators of salt sensitivity (Javed et al., 2021). Additionally, the sensitivity of crop species and genotypes also affects the extent of growth loss caused by salinity (Mühling and Läuchli, 2002). Generally, during the osmotic phase of salt stress, plant growth is drastically reduced (Munns and Tester, 2008). Compared to the NaCl treatment alone, the addition of Ca_2_SiO_4_ with NaCl resulted in a 30% and 24% increase in the fresh biomass of Iqbal and Jalal older leaves, while in younger leaves of Iqbal and Jalal, this increase was 26% and 22% (**Figures 1A, B**). Excessive Na^+^ accumulation in plant tissues, especially in the shoot, can be toxic as it disrupts cellular processes, interferes with nutrient uptake, and affects overall plant health therefore the reduction of sodium influx is important for plant growth and health. One of the key ways Si can influence Na^+^ movement is by forming a physical barrier in the root tissues. Si is taken up by plant roots and deposited in the form of silicon dioxide (SiO_2_) within the cell walls. This deposition creates a physical barrier that reduces the apoplastic movement of Na^+^ ions along the cell walls. As a result, Na^+^ ions are likely less to reach the plant’s vascular system, reducing their transport to the shoot. Si can contribute to improved crop plant growth by normalizing high salt concentrations and changing the efflux movement of Na^+^ from salt-stressed plant roots (Hussain et al., 2019). The results also indicated that the increase in fresh biomass with the introduction of Ca_2_SiO_4_ along with NaCl was 33% and 40% in the stem and 19% and 17% in the roots of Iqbal and Jalal, respectively (**Figures 1C, D**). Si treatment considerably reduces the risk associated with salinity since it helps to balance morphophysiological traits, mineral nutrition, and antioxidant defense mechanisms (Hassanvand et al., 2019). Si application can have multiple beneficial effects on plant physiology in salt-stressed environments, including decreased Na^+^ inflow, increased antioxidant defense system activity, improved photosynthetic rate-related leaf ultrastructure, and improved plant growth (Khorasaninejad and Hemmati, 2020).

Salinity stress impairs plant cell physiology by altering plant water relations, deteriorating membrane integrity, and reducing chlorophyll concentration. Compared to the control, the addition of NaCl resulted in a 22% and 33% reduction in the RWC of older and younger leaves of the Iqbal variety, while this reduction was 37% and 42% in older and younger leaves of the Jalal variety, respectively (**Table 1**). The RWC was noticeably reduced by the rising trend of NaCl in the growth media, which led plants to take up less water (Kumari et al., 2017). When compared to NaCl treatment, the supplementation of Ca_2_SiO_4_ with NaCl treatment resulted in 14.5% and 21% increases in RWC in older and younger leaves of the Iqbal variety, while 11.8% and 18.8% increases were noticed in older and younger leaves of the Jalal variety, respectively (**Table 1**). Si nutrition significantly increased the water content of the plant by enhancing the growth and capability of the roots to absorb more water from the growth medium and transfer it from the roots to the shoots, regulating the transpiration rate with stomatal conductance, regulating water potential and balancing osmotic adjustment (Wang et al., 2015). The increase in water content due to Si application is a consequence of the way Si interacts with the structural components of the plant, especially the cell walls. Si helps plants conserve water by reducing transpiration rates, regulating stomata behavior, and maintaining favorable water potential. Together, these mechanisms contribute to improved water retention and overall water management in plants, which is important for their health, especially under conditions of abiotic stress like high salinity. Earlier, beneficial impacts of Si application were suggested during salt and drought stress due to the maintenance of plant−water content and increased plant chlorophyll content (Maghsoudi et al., 2016). In comparison to SPAD values of maize in the NaCl treatment, SPAD values were found to be significantly higher when Ca_2_SiO_4_ was added along with NaCl in older and younger leaves of both Iqbal and Jalal varieties (**Table 1**). Earlier study (Zhu et al., 2019) elaborated that the accumulation of salts in plant leaves showed a significant impact on the chlorophyll content and photosynthetic activities of plants. Under certain stresses, photosynthesis is reduced, and necessary nutrients are more difficult to absorb, which causes a scarcity of critical metabolites (Riaz et al., 2021). Chlorosis begins to develop in leaves under severe salt stress, and with prolonged exposure, leaves begin to fall due to the induction of metabolic changes, disruption of hormonal regulation at the molecular level, stomatal closure, and alteration in the production of pigments essential to plant survival, such as chlorophyll, which ultimately leads to a decrease in chlorophyll contents (Qu et al., 2012). It was suggested in an earlier investigation (Acosta-Motos et al., 2015) that plant physiological attributes, such as the rate of transpiration, rate of photosynthesis, and opening and closing of stomata, are significantly affected as a result of high Na^+^ build-up in shoots, which can ultimately lead to reduced growth and later result in cell death.

### 4.2 Influence of salinity on the concentration of total ions in maize leaves

Plant salinity tolerance is largely dependent on maintaining the appropriate balance between ions and their optimal concentration. The increased uptake and buildup of Na^+^ is principally responsible for the damage to plant cells and the resulting growth retardations in crops subjected to salt stress (Qadir et al., 2017). The sensitivity of crop species and genotypes depends on the level of growth reduction caused by salinity (Shahzad et al., 2016). The current study revealed that older leaves had higher Na^+^ concentrations than younger leaves in both Iqbal and Jalal varieties under NaCl treatment; however, compared to the Iqbal variety, the Jalal variety presented a 27% increase in Na^+^ concentrations in both older and younger leaves (**Figures 2A, B**). Jalal variety’s higher Na^+^ concentrations suggest their potential toxicity and the need for mechanisms to regulate Na^+^ concentration and transport activities, which otherwise can inhibit various physiological processes and impaired plant growth. For many crop plant species, Na^+^ is more important than Cl, as reported in earlier studies, which has been linked to ion-specific damage caused by salt stress (Tester and Davenport, 2003). Compared to NaCl alone, a significantly decreased Na^+^ concentration, which was 27.1% in Iqbal and 17.6% in Jalal older leaves and 28.4% in Iqbal and 16% in Jalal younger leaves, was noticed when Ca_2_SiO_4_ was added to NaCl (**Figures 2A, B**). Earlier study (Flam-Shepherd et al., 2018), hypothesized that Si influences plant Na^+^ flux by regulating bypass flow, and by binding more Na^+^ to its surface, it may enhance plant growth (Khan et al., 2020). Soil salinity is involved in nutrient availability, competitive uptake, and transport within plants; as a result, it can cause nutritional imbalance. Certain nutrients such as K^+^, Mg^2+^, and Ca^2+^ uptake may be reduced because of the imbalance in nutrients and the competitive uptake of Na^+^ and Cl^−^ in a saline environment. The important regulator of cell homeostasis is K^+^, as within plant cells it plays critical roles by ensuring normal physiological functions such as osmotic adjustment, turgor generation, cell expansion, enzyme activation, pH homeostasis, and regulation of membrane electric potential (Hurtado et al., 2021) that are important for overall health of plant. Therefore, an optimal Na^+^/K^+^ ratio is crucial for various metabolic functions related to K^+^ as mentioned above. An increased Na^+^/K^+^ ratio as a result of higher Na^+^ concentration and reduced K^+^ concentration under high salt concentrations can result in a range of physiological and biochemical changes in the plant, including reduced growth, photosynthesis, and nutrient uptake (Noreen et al., 2017). In current study, a negative correlation was found between Na^+^ concentration and K^+^, Ca^2+^ and Mg^2+^ concentrations in different fractions *i.e.*, in total shoot, apoplast and symplast of *Zea mays* L. (**Figure 7**). Essential nutrients, e.g., Ca^2+^, K^+^, and Mg^2+^, uptake can be hampered due to excessive salt accumulation, which can affect functions related to plant metabolism and essential enzymes, resulting in osmotic imbalance and reduced plant growth (Ahmad et al., 2019). An important factor in determining the salt tolerance of plants can be calcium, as it is crucial for processes that maintain the structural and functional integrity of plant cell membranes, by stabilizing cell wall structures, controlling ion transport and selectivity, and regulating ion-exchange behavior as well as the activities of cell wall enzymes. However, the nature of these responses can be different according to the genotype of the plant (Hadi and Karimi, 2012). In the current study, supplementation with Ca_2_SiO_4_ along with NaCl consistently improved K^+^, Ca^2+^, and Mg^2+^ concentrations compared to NaCl treatment alone in both the Iqbal and Jalal varieties (**Figures 2A, B**). In comparison to non-Si-treated plants, Si-treated plants were able to sustain K^+^ concentration under salt stress (Etesami and Jeong, 2018). Increased H-ATPase pumps in the root plasma membrane can be attributed to the enhanced K^+^ concentration under Si application. In an earlier investigation (Naveed et al., 2020), it was suggested that Ca^2+^ might limit the uptake of Na^+^ in roots; as a result, increased Na^+^ retrieval from shoots can contribute to elevated K^+^ accumulation, ion homeostasis, and osmotic adjustment as well as a decreased Na^+^/K^+^ ratio under salt stress.

### 4.3 Ionic concentration in the apoplastic and symplastic fractions of maize leaves under salinity stress

The subcellular ions in maize leaves under salinity stress have only been the subject of a small number of investigations. The main cause of nutritional imbalances is higher accumulation of salt concentrations in various plant tissues. The accumulation of Na^+^ in tissue after a plant’s continuous exposure to salt and from the root export of Na^+^ to the shoot can become greater than the capacity of the individual cells to compartmentalize the ions into the vacuole. In this case, the salt ions will build up either in the cytoplasm or in the apoplastic fraction (Volkmar et al., 1998), as found in the current study, which can lead to metabolic toxicity and/or osmotic imbalance. Na^+^ ions tend to accumulate in plant cells, leading to an intracellular and extracellular ion imbalance. High Na^+^ can disturb the cell ability to maintain turgor pressure, it can result in reduced turgor pressure and potential plasmolysis which can cause osmotic stress within plant cells and can ultimately affect cell expansion and overall plant growth. Excessive Na^+^ ions also interfere with the activity of enzymes that play pivotal roles in various metabolic pathways and hindrance of essential biochemical reactions (Yang and Guo, 2018). The soluble Na^+^ concentration in the AWF was significantly higher in both older (36.1 mM) and younger (29.6 mM) leaves of the Jalal variety under NaCl treatment (**Figures 3A**, **4A**). These findings (*i.e.*, high Na^+^ concentrations in the apoplast) can result in osmotic inequality, which promotes cellular dehydration and turgor loss as suggested previously by Flowers et al. (1991), Moreover, in the current study, the soluble Na^+^ concentration in the apoplast of older leaves was found to be significantly greater in the salt-sensitive variety under NaCl treatment, which was 42.4% higher when compared to the Na^+^ concentration in the salt-tolerant variety under the same treatment. This suggest that the sensitivity of the cultivars against salt stress can be determined by their inability to control total Na^+^ flux to the shoot; as a result, higher xylem import of ions can be the cause of increased Na^+^ accumulation, especially in the sensitive maize cultivar, as Na^+^ is taken up by the root and subsequently transported to the xylem with the help of other transporters and channels. As suggested in an earlier investigation (Keisham et al., 2018), nonselective cation channels are involved in the uptake of Na^+^ by plant roots. If Na^+^ loading in symplastic or apoplastic pathways is not controlled, it can eventually reach the shoots via the xylem, where the unavoidable Na^+^ influx triggers a fast depolarization of the plasma membrane (Flowers et al., 2015), which can cause the cell membrane in plant shoots to leak several essential nutrients (Khan et al., 2020).

Dehydration of leaf cells caused by xylem Na^+^ entering the leaf apoplast, causing osmotic stress (*i.e.*, as this causes water to move from the cytoplasm to the apoplast, resulting in cellular dehydration and turgor loss), was previously thought to be the cause of reduced shoot growth, especially in the initial phase of salt stress. Earlier, it was hypothesized that variations in the time it takes for salt concentration in leaf vacuoles to reach its maximum level among genotypes are related to variations in salt tolerance (Munns, 1993). This process of high salt buildup may be facilitated by the avoidance of the Na^+^ concentration during salt stress in the expanding leaf apoplast. Interestingly, the soluble Na^+^ concentration in the AWF was significantly reduced in both varieties with the addition of Ca_2_SiO_4_ along with NaCl (**Figures 3A**, **4A**). Previously (Rogalla and Römheld, 2002), it was suggested that Si can mainly be deposited in the apoplast of the leaves, which proposes that there is less space available for Na^+^ accumulation in the apoplast of leaves. Metabolic activities might have benefited from calcium ions since they may compete with Na^+^ ions for the membrane binding sites and can also protect the cell membrane from the damaging effects of salinity. Higher apoplastic Na^+^ concentrations in the older leaves compared to younger leaves of both varieties (**Figures 3A**, **4A**) suggested that older leaves show necrosis first and result in cell death of the leaves. The elevated Na^+^ concentrations under salinity stress hinder Ca^2+^, K^+^, and Mg^2+^ transport into leaf cells, which may be the cause of the disruption of plant metabolism and decreased plant growth. Numerous studies have revealed that cations such as Mg^2+^, K^+,^ or Ca^2+^ have poor selectivity as a result of Na^+^ toxicity in many plants (Qadir et al., 2017; Kumar et al., 2019). In the current investigation, the increase in Na^+^ concentration corresponds nicely with the results of K^+^ concentration (**Figures 3A**, **4A**). Older leaves presented a higher concentration of K^+,^ and simultaneously, younger leaves showed a low K^+^ concentration. This indicates a possible competition effect between sodium and potassium for their uptake at the plasma membrane level (Munns and Tester, 2008). Sodium which reach the plasma membrane of a cell cause the membrane to depolarize and the K^+^ outward rectifier channels to open. This results in a loss of K^+^ from the cell (Pravin et al., 2016). The permeability of cell membrane increases significantly as salinity increases and is also reported to correlate with potassium (K) ions, osmotic adjustment, osmotic potential, and relative water contents. Genotypes can be characterized as tolerant or sensitive following varying patterns in cell membrane permeability, as in a saline condition, genotypes with salt sensitivity present marked alterations while salt-tolerant genotypes exhibit only minor modifications (Farooq and Azam, 2006). According to an earlier study (Raddatz et al., 2020), K^+^ transporters (HKT family) can promote Na^+^ uptake because K^+^ and Na^+^ have similar chemical characteristics, such as having an equal ion radius, which results in identical transport characteristics of these two ions and causes uncontrolled uptake of Na^+^. Furthermore, plasma membranes’ integrity and functionality, along with cell wall extensibility, depend on a sufficient amount of Ca^2+^ (Wei et al., 2003); therefore, it is possible that in the present study, the significantly higher Ca^2+^ concentration with the addition of Ca_2_SiO_4_ along with NaCl treatment in the leaf apoplast and symplast of the salt tolerant variety (**Figure 3C**) might have contributed to the improved leaf expansion. As the xylem is involved in the transport of calcium, increased RWC, as seen in the current investigation of stressed plants treated with Si (**Table 1**), is one explanation for the better Ca^2+^ transport in Si-treated plants under salt stress (Hattori et al., 2005). The length of time needed to reach its highest concentration for salt in the leaf vacuoles may be connected to variations in salt tolerance. Under stress situations, this process of excessive salt buildup may help to avoid harmful ion concentrations in the expanding leaf apoplast. Salt flux into the symplast is encouraged by the moderately loaded maize apoplast during a protracted salt treatment, resulting in increased concentrations initially in the vacuole and later in the symplast. In summary, uncontrolled Na^+^ loading in apoplastic or symplastic pathways can eventually reach the shoots via the xylem, where the unavoidable Na^+^ influx can cause the cell membrane in plant shoots to leak several essential nutrients.

In the current study, the soluble Na^+^ concentration in the apoplast of older leaves was found to be significantly greater in the salt-sensitive maize variety under NaCl treatment, which was 42.4% higher when compared to the Na^+^ concentration in the salt-tolerant variety under the same treatment. The accumulation of Na^+^ in the extracted symplastic and apoplastic fluids suggests that plant failure to regulate the total salt flux into the apoplastic fraction of the leaf and, from there, into the cells can be the cause of maize salt sensitivity. However, partial amelioration and increased salinity tolerance of maize with the addition of silicon under salt stress might be attributable to increased water contents, decreased apoplastic Na^+^ loading, and intracellular ionic homeostasis. These findings are crucial for understanding partly why salt-sensitive varieties perform less than salt-tolerant ones. Further studies can compare salt tolerance with other cultivars especially dicots, which could serve as important targets for improving salt stress tolerance.

## Data availability statement

The original contributions presented in the study are included in the article/supplementary material. Further inquiries can be directed to the corresponding authors.

## Author contributions

MZM: Data curation, Formal analysis, Investigation, Methodology, Writing – original draft. HAO: Funding acquisition, Visualization, Writing – review & editing. RA: Conceptualization, Methodology, Resources, Software, Validation, Writing – review & editing. MKG: Funding acquisition, Software, Visualization, Writing – review & editing. MS: Conceptualization, Funding acquisition, Methodology, Project administration, Resources, Software, Supervision, Validation, Writing – review & editing. AMA: Conceptualization, Funding acquisition, Methodology, Project administration, Software, Validation, Visualization, Writing – review & editing.
